# Night-shift work is associated with peripheral clock gene dysregulation and circadian transcriptional network remodeling in female healthcare workers

**DOI:** 10.3389/fendo.2026.1934310

**Published:** 2026-09-24

**Authors:** Silvia Vivarelli, Luca Falzone, Francesca Simona Fiorino, Federica Giambò, Massimo Libra, Concettina Fenga

**Affiliations:** 1 Department of Biomedical and Dental Sciences, Morphological and Functional Imaging, Section of Occupational Medicine, University of Messina, Messina, Italy; 2 Department of Biomedical and Biotechnological Sciences, University of Catania, Catania, Italy; 3 Vita-Salute San Raffaele University, Milano, Italy

**Keywords:** biomarkers, chronodisruption, circadian rhythm, clock genes, gene expression profiling, healthcare workers, night-shift work, occupational health

## Abstract

**Background:**

Night-shift work disrupts the circadian–melatonin axis through chronic light-at-night exposure and has been associated with metabolic, inflammatory, cardiovascular, and cancer-related disorders, but peripheral molecular signatures of occupational chronodisruption remain incompletely characterized. Female healthcare workers (HCWs), frequently exposed to prolonged night-shift schedules, represent a relevant study population. In a previous computational study, we identified a deregulated circadian gene panel (CLOCK, TEF, PER1, and PER3) suggestive of transcriptional remodeling in exposed workers. Here, we experimentally validated this signature and investigated the non-canonical circadian transcription factor YY1.

**Methods:**

Ninety-three female HCWs (47 day-shift, DSW; 46 night-shift workers, NSW) participated in the study. Peripheral blood expression of CLOCK, TEF, PER1, PER3, and YY1 was quantified by RT-qPCR. Associations with shift-work status and exposure metrics were evaluated using multivariable regression models adjusted for demographic, reproductive, lifestyle, and sleep-related covariates. Correlation, principal component, and clustering analyses were used to characterize transcriptional organization.

**Results:**

Compared with DSW, NSW exhibited significantly lower expression of CLOCK (p = 0.0362), TEF (p < 0.0001), and PER1 (p = 0.0003), whereas PER3 expression was unchanged (p = 0.7978). TEF and CLOCK expression progressively declined with longer duration and higher cumulative night-shift exposure. TEF showed excellent in-sample discrimination between groups (AUC = 0.925), while a four-gene model achieved an AUC of 0.940. YY1 expression was reduced in NSW (p = 0.0019), decreased across long-duration and high-exposure categories, and further improved multigene classification performance (AUC = 0.976). Network analyses revealed disrupted coordination among CLOCK, TEF, and PER1, whereas PER3 maintained stable expression despite reduced integration within the transcriptional network.

**Conclusions:**

Night-shift work exposure is associated with coordinated downregulation of CLOCK, TEF, and PER1, altered transcriptional network organization, and reduced YY1 expression. While PER3 expression is preserved, its network connectivity is selectively weakened in exposed workers. These findings identify peripheral transcriptional signatures that may provide a basis for future biomarker development and exposure characterization and support further investigation of chronobiology-driven preventive strategies within a precision medicine framework.

## 1 Introduction

Circadian rhythms are endogenous biological oscillations that coordinate a broad range of physiological processes, including endocrine signaling, immune responses, metabolism, and gene expression (1). These rhythms are regulated by a highly conserved molecular clockwork composed of core clock genes, such as CLOCK, BMAL1, PER, and CRY, which generate self-sustained transcriptional–translational feedback loops that synchronize cellular and systemic functions with the external light–dark cycle (2, 3). Maintaining alignment between endogenous circadian timing and environmental cues is essential for physiological homeostasis and health (4).

Night-shift work is a major source of circadian disruption in modern societies. By imposing wakefulness during the biological night and sleep during the biological day, it induces chronic misalignment between internal rhythms and behavioral cycles (5, 6). Mechanistically, exposure to artificial light at night suppresses pineal melatonin secretion, the principal neuro-endocrine signal coordinating peripheral circadian oscillators (7). Such disruption might lead to several adverse health outcomes, including metabolic disorders, cardiovascular disease, immune dysfunction, and cancer (7–11). Consistent with these observations, the International Agency for Research on Cancer (IARC) has classified night-shift work as probably carcinogenic to humans (Group 2A) (12). In particular, long-term night-shift work has been associated with an increased risk of breast cancer, although the biological pathways linking circadian dysregulation to disease development remain only partially understood (13).

Female healthcare workers constitute a particularly relevant population in which to investigate these effects because they are frequently exposed to rotating and night-shift schedules over prolonged periods (14–17). Repeated exposure may amplify health consequences (10, 18). Moreover, biological factors, including hormonal fluctuations across the reproductive lifespan and reproductive aging, may influence circadian regulation and individual vulnerability (19, 20).

At the molecular level, night-shift work has been associated with alterations in peripheral clock gene expression and broader transcriptional programs involved in metabolic, inflammatory, and stress-response pathways (21–24). In previous computational analyses, we identified a set of deregulated circadian genes (CLOCK, TEF, PER1, and PER3) linked with breast cancer susceptibility among night-shift workers, supporting the hypothesis that chronic circadian disruption may induce persistent transcriptional remodeling (25). Beyond established clock components, increasing evidence suggests that circadian adaptation and maladaptation are influenced by non-core regulatory factors that integrate environmental and cellular signals with circadian transcriptional networks (26, 27). These include regulators such as SIRT1, AMPK, STAT3, and HIF-1α, which link energy metabolism, inflammation, and cellular stress responses to clock function (28–31). Among these non-canonical regulators, Yin Yang 1 (YY1) is of particular interest because of its dual role in transcriptional regulation and chromatin remodeling, processes that may contribute to the persistent reorganization of gene expression under chronic circadian stress (32). Experimental and computational studies have implicated YY1 in circadian transcriptional programs and interactions with molecular clock components, supporting its investigation as a candidate non-canonical regulator of peripheral circadian responses (33–36).

Peripheral blood cells retain functional circadian machinery and provide a minimally invasive system for studying systemic circadian regulation in humans (37–43). However, previous studies have been limited by small cohorts, restricted gene panels, and heterogeneous findings regarding the extent of transcriptional disruption associated with night-shift exposure (44). Importantly, the occupational circadian response has been less extensively investigated beyond canonical clock genes, as well as at the level of transcriptional network organization.

To address these gaps, we investigated peripheral blood expression of selected core clock genes and the non-canonical circadian regulator YY1 in female healthcare workers with varying levels of night-shift exposure. We further examined associations between these molecular parameters and occupational exposure metrics, including total years of employment, cumulative number of night shifts, and recent shift activity. By integrating differential gene expression analysis and network-based approaches, we aimed to identify molecular signatures associated with occupational chronodisruption with potential translational relevance for biomarker development and exposure characterization.

## 2 Methods

### 2.1 Study design and population

This cross-sectional study was conducted within the occupational health surveillance program of the AOU G. Martino and included female healthcare workers (HCWs). A total of 93 HCWs provided written informed consent and participated in the study. Participants were classified according to their current work schedule as exclusively day-shift workers (DSW; n = 47) or current night-shift workers (NSW; n = 46). DSW had no history of previous night-shift work, whereas all NSW were currently assigned to a work schedule that included night shifts at the time of study enrollment. Eligible participants were aged 37–66 years and had been employed under the same shift regimen for at least 6 months. Exclusion criteria included pregnancy, acute or chronic conditions potentially affecting circadian homeostasis.

Night-shift work was defined according to Directive 2003/88/EC as any schedule including ≥3 h of work performed between 00:00 and 05:00 h, in accordance with institutional shift organization criteria. Among NSW, the extent and temporal pattern of night-shift exposure were characterized using duration of night-shift work, cumulative lifetime number of night shifts, and recent night-shift activity. Duration of night-shift work was classified as short-term (1–12 years) or long-term (13–35 years), while cumulative exposure was dichotomized as low (<1000 shifts) or high (≥1000 shifts), corresponding approximately to the 40th percentile of the distribution. Recent night-shift activity was defined as having performed at least one night shift during the six months preceding study enrollment and was recorded as a separate measure of recent occupational exposure. Five NSW participants with a history of breast cancer were evaluated in exploratory subgroup analyses.

### 2.2 Occupational health surveillance and clinical assessment

Participants underwent standardized occupational and clinical evaluations conducted by trained occupational physicians between January 2024 and December 2025. Clinical, reproductive, and lifestyle data were collected to account for established determinants of circadian variability.

Medical and reproductive history, including menopausal status and parity, was obtained through structured interviews. Lifestyle variables included smoking status, alcohol consumption, physical activity, sleep duration, and self-reported sleep quality.

Anthropometric measurements were performed using standardized procedures. Body mass index (BMI) was calculated as weight (kg)/height (m²).

### 2.3 Blood sampling and laboratory testing

Venous blood samples were collected in the morning (07:30–09:00 h) after an overnight fast of at least 8 h under standardized conditions to minimize pre-analytical variability. Whole-blood samples were centrifuged at 2000 × g for 10 min, and plasma, buffy coat, and erythrocyte fractions were separated, aliquoted, and stored at −80 °C until analysis. Prior to storage, buffy coat samples were diluted with physiological saline to a final volume of 1 mL.

### 2.4 Clock gene expression analysis

Total RNA was extracted from buffy coat samples using a combined TRIzol/PureLink™ RNA Mini Kit (Invitrogen, Thermo Fisher Scientific). RNA concentration and purity were assessed using a NanoDrop 1000 spectrophotometer (Thermo Fisher Scientific). RNA samples with an A260/A280 ratio 1.7–2.1 were considered suitable for downstream RT-qPCR analysis.

Gene expression was quantified using one-step RT-qPCR (QuantiTect SYBR Green RT-PCR Kit, Qiagen). Each 25 µL reaction contained SYBR Green Master Mix, RT Mix, gene-specific primers, RNase-free water, and RNA template. All samples were run in technical duplicates using a QIAquant 95 5plex thermocycler (Qiagen). Commercially available QuantiTect Primer Assays (Qiagen; GeneGlobe), consisting of pre-designed and experimentally validated primer assays, were used for target amplification. Amplification specificity was assessed by melting-curve analysis, with only assays showing a single specific amplification product included in the analysis. Together, the validated assay design and melting-curve analysis ensured target-specific amplification. Gene-specific primers are reported in **Table 1**.

**Table 1:** Primer and assay characteristics of target and reference genes used for RT-qPCR analysis.

Gene	Gene name	Entrez ID	GeneGlobe ID	Amplicon (bp)	Amplified exons	Detected transcripts
CLOCK	clock circadian regulator	9575	QT00054481	71	21/22	NM_004898, NM_001267843, XM_005265787, XM_006714054
TEF	thyrotrophic embryonic factor	7008	QT00032732	64	2/3	NM_001145398, NM_003216
PER1	period circadian clock 1	5187	QT00069265	118	17/18	NM_002616, XM_005256689
PER3	period circadian clock 3	8863	QT00097713	117	20/21	NM_016831, NM_001289861–NM_001289864
YY1	YY1 transcription factor	7528	QT00052738	135	2/3/4	NM_003403
ACTB (housekeeping gene)	actin beta	60	QT00095431	146	3/4	NM_001101

Mean Ct values from technical duplicates were used for all downstream analyses. Relative gene expression was calculated using the comparative Ct (2^−ΔΔCt^) method with ACTB as endogenous housekeeping gene (45–47). For each target gene, the mean ΔCt of DSW was used as calibrator. Expression levels were expressed as log_2_ fold change (log_2_FC), corresponding to −ΔΔCt.

A composite Clock Gene Expression Score (CGES) was calculated as the mean of standardized z-scores of CLOCK, PER1, PER3, and TEF (formula used: CGES = [z(CLOCK) + z(PER1) + z(PER3) + z(TEF)]/4).

### 2.5 Statistical analyses

Statistical analyses were performed using GraphPad Prism (version 9.0) and additional web-based platforms as specified below. Normality of data distribution was assessed using the Kolmogorov–Smirnov test. Primary outcomes were peripheral blood expression levels of CLOCK, TEF, PER1, and PER3, together with CGES. Secondary outcomes included YY1 expression and BC subgroup exploratory analysis.

Continuous variables are presented as mean ± standard deviation or median (interquartile range), as appropriate, while categorical variables are reported as frequencies and percentages. Missing data were handled using complete-case analysis without imputation. Between-group comparisons (DSW vs NSW and exposure subgroups within NSW) were performed using the Mann–Whitney U test or Kruskal–Wallis test followed by Dunn’s *post hoc* test, as appropriate. Categorical variables were analyzed using chi-square tests.

Receiver operating characteristic (ROC) curve analyses were used to evaluate the discriminatory ability of individual genes and multigene models. Area under the curve (AUC), 95% confidence intervals (CIs), and p-values were reported. Because the analyses were exploratory, reported AUC estimates were considered apparent (in-sample) discrimination estimates and were not internally or externally validated.

Multivariable linear regression models were fitted separately for each target gene to assess associations with demographic, reproductive, lifestyle, and sleep-related covariates. Models included age, BMI, menopausal status, parity, smoking status, alcohol consumption, physical activity, sleep duration, and sleep quality. Regression coefficients (β), p-values, and R² values were reported.

Multivariable logistic regression models were used to evaluate the ability of gene expression profiles to discriminate between DSW and NSW and between exposure categories (duration, cumulative, and recent exposure). Odds ratios (ORs), 95% CIs, likelihood ratio tests, accuracy, and ROC curves were reported.

Spearman correlation analyses were performed with Benjamini–Hochberg false discovery rate (FDR) correction to account for multiple testing. Correlation matrices were converted into distance matrices and compared using Mantel tests with 10,000 permutations. Analyses were performed using the web-based statistical platform ChiPlot (48).

Principal component analysis (PCA) and hierarchical clustering were performed using ClustVis (49). Data were mean-centered and scaled to unit variance. PCA included all clock gene expression variables, with the first three components retained for visualization. Hierarchical clustering used Euclidean distance with complete linkage. Heatmaps were used to visualize clustering patterns across groups. All tests were two-sided, and statistical significance was set at p < 0.05.

## 3 Results

### 3.1 Night-shift work is associated with selective dysregulation of peripheral clock genes expression

The study included 93 female healthcare workers, comprising 47 day-shift workers (DSW) and 46 night-shift workers (NSW). The two groups were comparable in terms of age (51.64 ± 8.72 vs. 51.00 ± 7.55 years, p = 0.707) and body mass index (24.26 ± 5.34 vs. 24.44 ± 4.47 kg/m², p = 0.857) (see **Supplementary Table 1**).

Among NSWs, 54% reported long-term night-shift exposure (13–35 years) and 57% had accumulated ≥1000 lifetime night shifts. Recent night-shift activity was documented in 20 of 46 NSW participants (43%), whereas 26 (57%) had not performed a night shift during the preceding six months. Among the 20 participants with recent activity, the number of night shifts ranged from 3 to 8, with a mean of 5.4 ± 1.1 shifts (**Supplementary Table 2**).

Clinical and lifestyle characteristics are summarized in **Supplementary Table 3**. Most variables did not differ significantly between groups, including parity, menopausal status, smoking habits, and physical activity (all p > 0.05). Alcohol consumption was more frequently reported among NSW than DSW (76% vs. 55%, p = 0.035). Sleep quality was poorer among NSW, with a higher prevalence of poor or very poor sleep satisfaction (54% vs. 40%, p = 0.004), whereas sleep duration showed a borderline reduction (5.76 ± 1.08 vs. 6.26 ± 1.31 h, p = 0.051).

Analysis of peripheral clock gene expression revealed significant differences between groups (**Figure 1A**). Compared with DSW, NSW exhibited lower expression of CLOCK (p = 0.0362), TEF (p < 0.0001), and PER1 (p = 0.0003). In contrast, PER3 expression did not differ significantly between groups (p = 0.7978).

**Figure 1:**
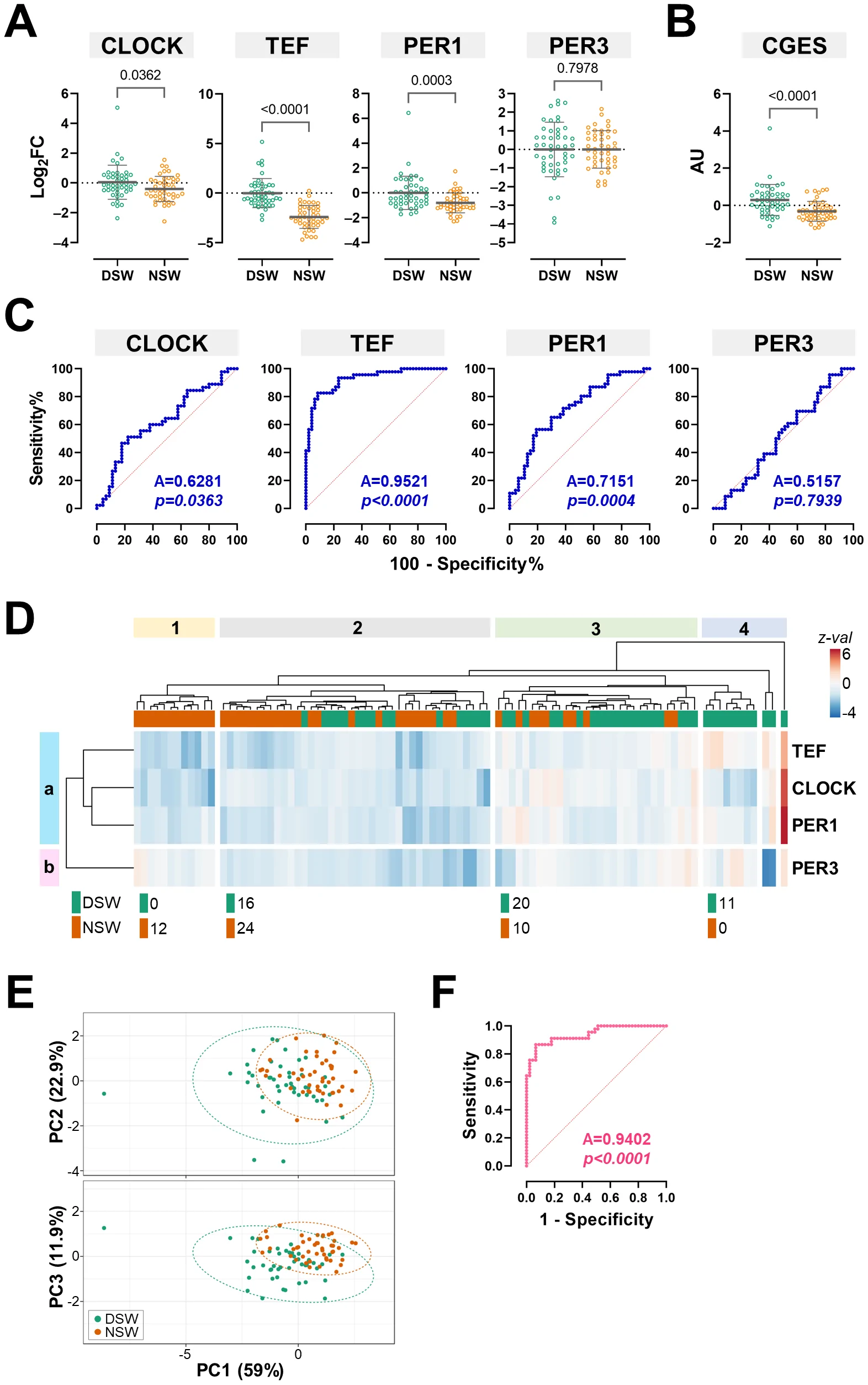
Circadian transcriptional profiling and predictive modeling of night-shift exposure in healthcare workers. **(A)** RT-qPCR-derived log2 fold-change (log2FC) expression of core clock genes (CLOCK, TEF, PER1, PER3) in day-shift workers (DSW; n = 47, green) and night-shift workers (NSW; n = 46, orange). Individual values are shown as dot plots; group means ± SD are indicated. Exact p-values for between-group comparisons are reported within each panel. **(B)** Clock Gene Expression Score (CGES, arbitrary units) in DSW and NSW. Data are presented as in **(A)**, with group means ± SD and exact p-value. **(C)** Receiver operating characteristic (ROC) curves evaluating the discriminatory performance of individual gene expression models (CLOCK, TEF, PER1, PER3) for classification of shift-work status. Area under the curve (AUC) values and corresponding p-values are reported. **(D)** Heatmap of standardized gene expression (z-scores) for CLOCK, TEF, PER1, and PER3 across individual participants. Expression levels are displayed on a blue-to-red scale (lower to higher expression). Columns represent participants. Hierarchical clustering identifies two gene modules (Cluster a: TEF, CLOCK, PER1; Cluster b: PER3) and four participant clusters (1–4), with proportions of DSW and NSW indicated per cluster. **(E)** Principal component analysis (PCA) based on the four clock genes. Score plots show partial separation between DSW (green) and NSW (orange), displayed as PC1 vs PC2 (top) and PC1 vs PC3 (bottom). Ellipses represent 95% confidence intervals. **(F)** ROC curve of the multivariable logistic regression model integrating all clock gene expression features for discrimination between DSW and NSW. AUC and p-value are reported. Statistical significance was defined as two-sided p < 0.05.

To obtain an integrated measure of circadian transcriptional activity, a composite Clock Gene Expression Score (CGES) was calculated from the standardized expression values of CLOCK, TEF, PER1, and PER3 (**Figure 1B**). NSW showed significantly lower CGES values than DSW (p < 0.0001), consistent with an overall reduction in peripheral clock gene expression.

### 3.2 Night-shift exposure signature in peripheral clock genes is robust to confounding and shows strong discriminatory performance

Multivariable linear regression models were used to assess whether differences in clock gene expression were independent of relevant clinical and lifestyle covariates (**Table 2**). The models showed low-to-moderate explanatory power for TEF (R² = 0.222; model p = 0.0098), PER1 (R² = 0.184; model p = 0.0405), and PER3 (R² = 0.171; model p = 0.0628), whereas CLOCK was not significantly explained by the included covariates (R² = 0.124; model p = 0.271). Among individual covariates, alcohol consumption was negatively associated with TEF expression (β = −0.836; p = 0.031). PER1 expression was positively associated with BMI (β = 0.057; p = 0.029) and smoking status (β = 0.351; p = 0.012). Other covariates were not significantly associated with gene expression (all p > 0.05). Overall, these findings indicate that variability in clock gene expression is only partially explained by classical demographic and lifestyle factors.

**Table 2:** Multivariable linear regression analysis of clock gene expression and clinical–lifestyle covariates.

Gene	Model R²	Model p	Age β (p)	BMI β (p)	Menopause β (p)	Pregnancies β (p)	Smoking β (p)	Alcohol β (p)	Sport β (p)	Sleep length β (p)	Sleep dissatisfaction β (p)
CLOCK	0.124	0.271	-0.010 (0.665)	0.044 (0.093)	0.156 (0.688)	-0.229 (0.093)	0.222 (0.083)	-0.030 (0.901)	-0.139 (0.562)	0.037 (0.754)	-0.141 (0.431)
TEF	0.222	**0.0098**	0.055 (0.155)	0.051 (0.189)	-0.802 (0.203)	-0.131 (0.549)	0.160 (0.435)	**-0.836 (0.031)**	-0.492 (0.205)	0.305 (0.107)	-0.332 (0.259)
PER1	0.184	**0.0405**	-0.016 (0.541)	**0.057 (0.029)**	0.447 (0.291)	-0.083 (0.572)	**0.351 (0.012)**	-0.282 (0.275)	0.235 (0.368)	0.187 (0.140)	0.027 (0.890)
PER3	0.171	0.0628	-0.019 (0.486)	-0.009 (0.740)	0.582 (0.203)	-0.282 (0.077)	0.252 (0.092)	-0.279 (0.317)	**0.646 (0.023)**	-0.028 (0.838)	-0.398 (0.064)

Values represent standardized regression coefficients (β) from multivariable linear regression models, with corresponding p-values reported in parentheses. Model R² and overall model p-values are shown for each gene. Statistically significant associations (p < 0.05) are reported in bold.

Receiver operating characteristic (ROC) curve analyses were then performed to evaluate the ability of individual genes to discriminate between DSW and NSW (**Figure 1C**). CLOCK showed modest discriminatory performance (AUC = 0.6281; 95% CI: 0.512–0.744; p = 0.0363), while PER1 showed fair performance (AUC = 0.7151; 95% CI: 0.611–0.819; p = 0.0004). TEF showed high apparent discriminatory performance (AUC = 0.9251; 95% CI: 0.873–0.977; p < 0.0001), whereas PER3 did not discriminate between groups (AUC = 0.5157; p = 0.7939).

Unsupervised hierarchical clustering of gene expression profiles showed partial separation between DSW and NSW (**Figure 1D**). At the gene level, CLOCK, TEF, and PER1 clustered together, while PER3 formed a separate branch. At the participant level, four clusters were identified, including one predominantly composed of NSW (n = 12) and one enriched in DSW (n = 11), with two mixed clusters containing both exposure groups.

Principal component analysis (PCA) supported these findings (**Figure 1E**). PC1 explained 59.0% of total variance and was driven primarily by PER1 (loading = 0.601), TEF (0.537), and CLOCK (0.508). PC2 explained 22.9% of variance and was dominated by PER3 (loading = 0.915). PC3 explained 11.9% of variance and reflected additional gene-specific variability. Score plots showed partial separation of NSW and DSW along PC1, consistent with a global shift in peripheral clock gene expression.

Finally, a multivariable logistic regression model integrating gene expression values confirmed the classification performance of the transcriptional signature (**Table 3**). TEF was negatively associated with NSW status (OR = 0.07; 95% CI: 0.018–0.192; p < 0.0001), while PER3 was positively associated with NSW status (OR = 3.06; 95% CI: 1.28–8.99; p = 0.022). CLOCK and PER1 were not independently associated with group status. The combined model showed excellent apparent discrimination (AUC = 0.9402; 95% CI: 0.894–0.987; p < 0.0001; **Figure 1F**), indicating that combined transcriptional profiles showed greater in-sample discrimination between DSW and NSW than individual genes.

**Table 3:** Multivariable logistic regression analysis of gene expression predictors of night-shift work status.

Gene	β (SE)	Odds Ratio (95% CI)	p-value	Interpretation
CLOCK	0.665 (0.606)	1.94 (0.63–6.99)	0.273	Not significant
TEF	-2.649 (0.597)	**0.07 (0.018–0.192)**	**<0.0001**	Strong inverse association (DSW-predictive)
PER1	-0.343 (0.671)	0.71 (0.19–2.75)	0.610	Not significant
PER3	1.117 (0.489)	**3.06 (1.28–8.99)**	**0.022**	Positive association (NSW-predictive)

Values represent regression coefficients (b) with standard errors (SE), odds ratios (OR), and 95% confidence intervals (CI) from multivariable logistic regression models. Statistically significant associations (p < 0.05) are reported in bold.

### 3.3 Peripheral clock genes expression is tightly coordinated and differentially structured across study variables and shift-work status

Relationships between peripheral clock gene expression, the composite Clock Gene Expression Score (CGES), and clinical–lifestyle variables were evaluated using Spearman correlation analysis and Mantel tests (see **Supplementary Table 4**, and **Figure 2A**).

**Figure 2:**
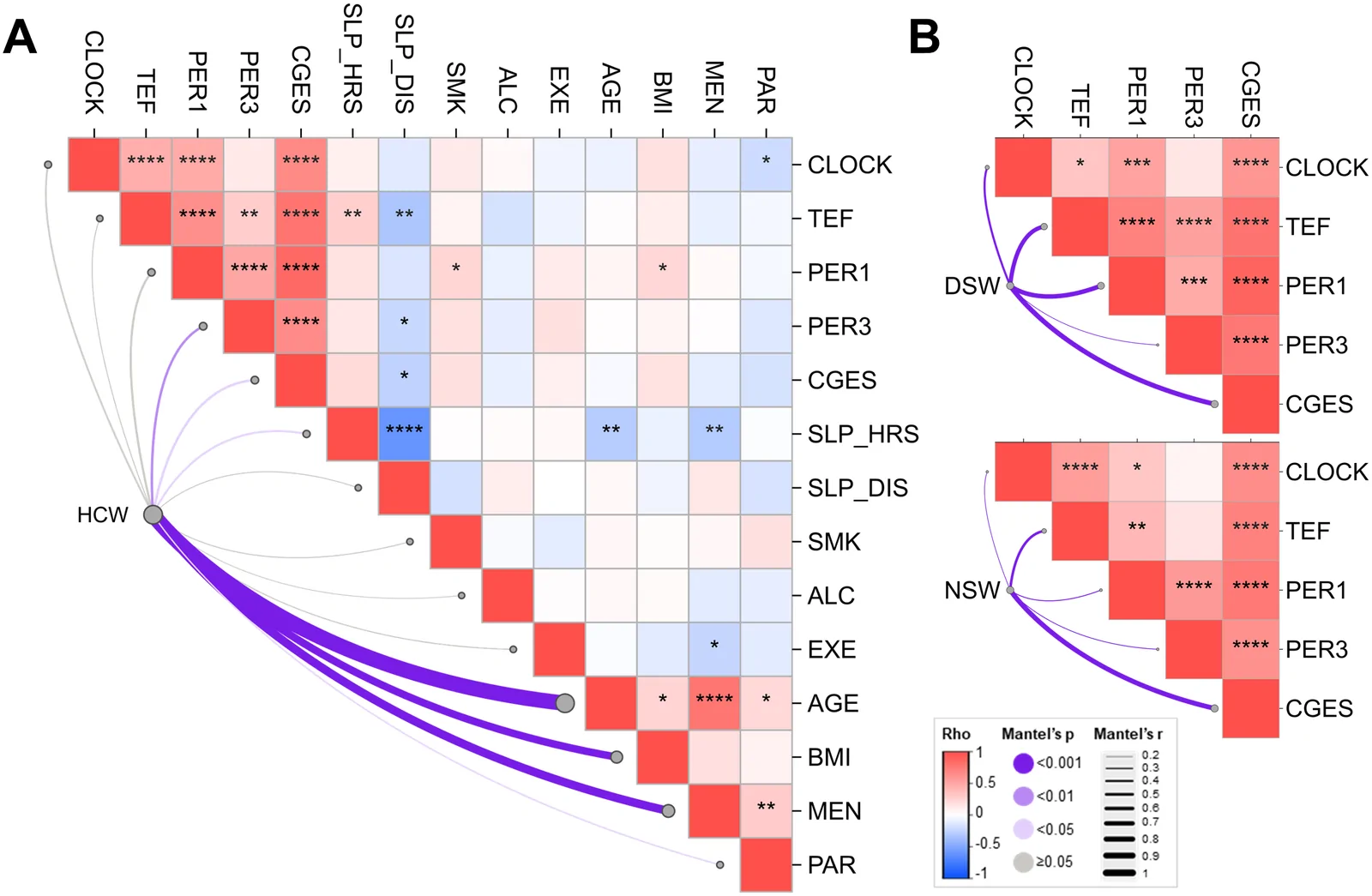
Correlograms of clock genes and behavioral, demographic, and anthropometric variables in healthcare workers. **(A)** Spearman correlation heatmap combined with Mantel network visualization in the overall cohort of healthcare workers (HCWs), including CLOCK, TEF, PER1, PER3, and the Clock Gene Expression Score (CGES), together with sleep duration (SLP_HRS), sleep dissatisfaction (SLP_DIS), smoking status (SMK), alcohol consumption (ALC), physical exercise (EXE), age (AGE), body mass index (BMI), menopausal status (MEN), and parity (PAR). **(B)** Stratified analyses in day-shift workers (DSW, upper panel) and night-shift workers (NSW, lower panel) for CLOCK, TEF, PER1, PER3, and CGES. Correlation coefficients (ρ) are represented using a continuous color scale ranging from blue (ρ = −1) through white (ρ = 0) to red (ρ = +1), indicating negative and positive associations, respectively. Statistically significant correlations are indicated by asterisks based on Benjamini–Hochberg false discovery rate (FDR)-adjusted p-values: *p < 0.05, **p < 0.01, ***p < 0.001, and ****p < 0.0001. Mantel network edges represent variable-level associations; edge thickness is proportional to Mantel r values, and edge color reflects statistical significance (dark purple: p < 0.001; medium purple: p < 0.01; light purple: p < 0.05; grey: p ≥ 0.05).

Strong positive correlations were observed among circadian transcripts. The strongest associations were found between TEF and PER1 (ρ = 0.64, p < 0.001), TEF and CGES (ρ = 0.80, p < 0.001), PER1 and CGES (ρ = 0.86, p < 0.001), and CLOCK and CGES (ρ = 0.67, p < 0.001). PER3 was also positively correlated with the other clock genes, although with lower correlation coefficients (ρ ranging from 0.13 to 0.51).

Correlations between clock gene expression and demographic or lifestyle variables were generally weaker. Sleep duration was positively correlated with TEF (ρ = 0.27) and CGES (ρ = 0.20), whereas sleep dissatisfaction was negatively correlated with TEF (ρ = −0.33) and CGES (ρ = −0.26) (all p < 0.05). No relevant correlations were observed for age, BMI, smoking status, alcohol consumption, or physical activity.

Mantel tests identified significant associations between the circadian expression matrix and several biological variables, including age (r = 0.90, p = 0.001), BMI (r = 0.42, p = 0.001), and menopausal status (r = 0.48, p = 0.001). In contrast, smoking status, alcohol consumption, and physical activity were not significantly associated with the overall correlation structure (all p > 0.05).

Stratification by shift-work status revealed differences in correlation patterns between groups (see **Supplementary Tables 5**, **S6**, and **Figure 2B**). In DSW, strong positive correlations were observed between CGES and PER1 (ρ = 0.89), TEF (ρ = 0.80), CLOCK (ρ = 0.60), and PER3 (ρ = 0.77) (all p < 0.001). Strong associations were also observed among individual genes, particularly between TEF and PER1 (ρ = 0.72). In NSW, CGES remained significantly correlated with TEF (ρ = 0.71), PER1 (ρ = 0.76), CLOCK (ρ = 0.65), and PER3 (ρ = 0.63) (all p < 0.001). However, several pairwise correlations were weaker than those observed in DSW. Notably, the correlation between CLOCK and PER3 was markedly reduced in NSW (ρ = 0.07).

Mantel test analyses showed significant clustering of circadian variables in both groups. Correlation strengths were generally higher in DSW (CGES r = 0.87; PER1 r = 0.85; TEF r = 0.83; all p = 0.001) than in NSW (CGES r = 0.80; TEF r = 0.66; PER1 r = 0.56; PER3 r = 0.55; all p = 0.001), indicating differences in the overall correlation structure according to shift-work status.

### 3.4 Duration and cumulative night-shift exposure are associated with differential peripheral clock genes expression profiles

Peripheral clock gene expression was further evaluated according to duration of night-shift work, cumulative lifetime exposure, and recent exposure status (**Figures 3A–C**). When stratified by duration of night-shift work, CLOCK expression was significantly lower in the long-exposure group than in the short-exposure group (p = 0.0220). TEF expression was also reduced among workers with longer exposure (p = 0.0125). In contrast, no significant differences were observed for PER1 (p = 0.4057) or PER3 (p = 0.4439) (**Figure 3A**).

**Figure 3:**
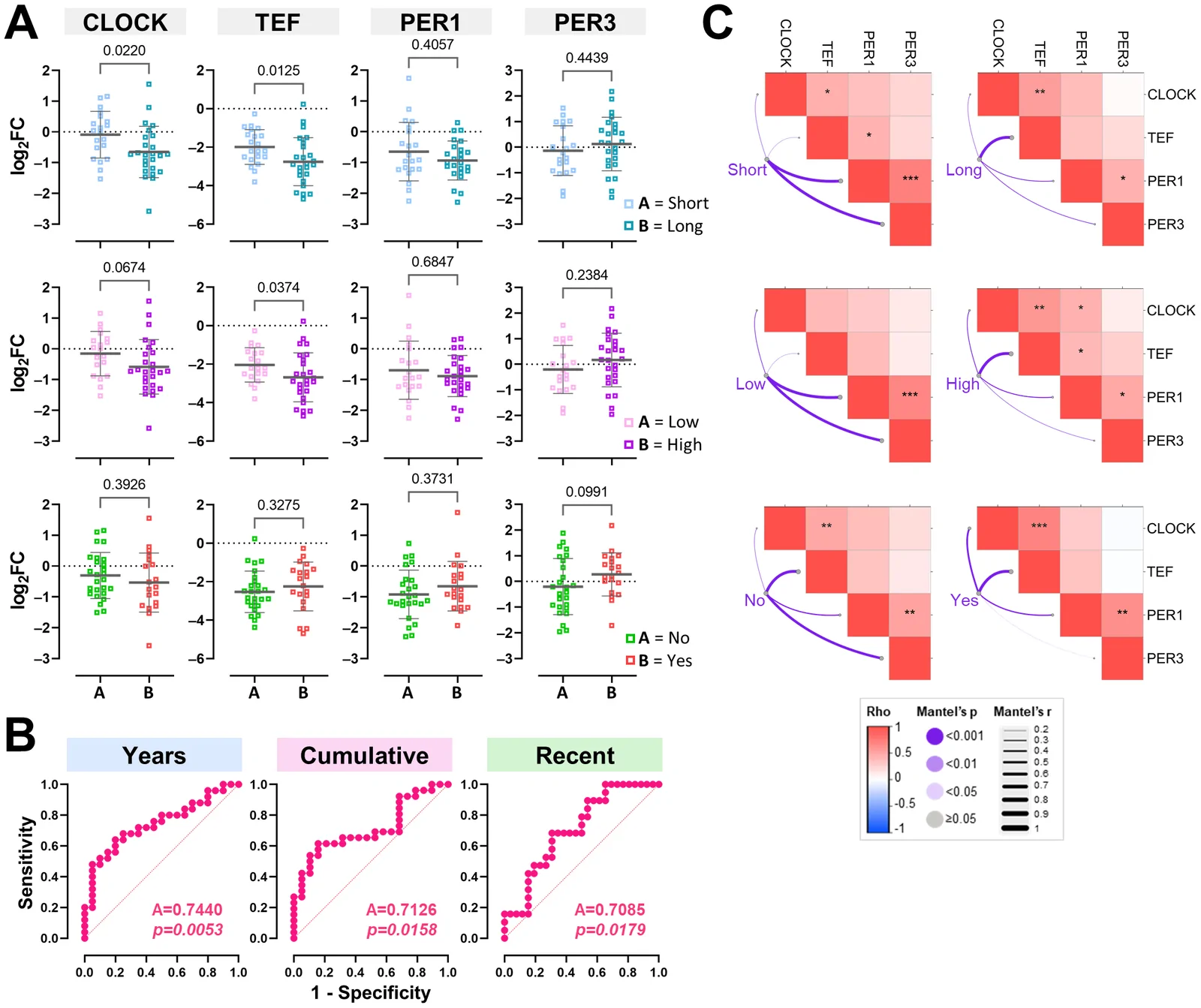
Clock genes expression and occupational exposure in night-shift workers. **(A)** Dot plots showing RT-qPCR-derived log2 fold-change (Log2FC) expression of core clock genes (CLOCK, TEF, PER1, and PER3) in night-shift workers (NSW; N = 46). Each dot represents an individual participant; mean ± SD are shown within each group, and exact p-values are reported for between-group comparisons. Analyses are stratified by exposure level: years of night-shift work (short: N = 21; long: N = 25), cumulative exposure (low: N = 20; high: N = 26), and recent exposure (no exposure: N = 26; exposure: N = 20). **(B)** Receiver operating characteristic (ROC) curves from multivariable logistic regression models assessing the predictive performance of circadian gene expression signatures for exposure categories, including years of exposure (left), cumulative exposure (center), and recent exposure (right). Area under the curve (AUC) values and corresponding p-values are reported. **(C)** Spearman correlation heatmaps and Mantel network analyses for CLOCK, TEF, PER1, and PER3 stratified by exposure category (top: short vs long years of exposure; middle: low vs high cumulative exposure; bottom: no vs yes recent exposure). Correlation coefficients (ρ) are displayed using a continuous color scale ranging from blue (ρ = −1) through white (ρ = 0) to red (ρ = +1). Statistically significant correlations are indicated by asterisks based on Benjamini–Hochberg false discovery rate (FDR)-adjusted p-values: *p < 0.05, **p < 0.01, and ***p < 0.001. Mantel network edges represent gene-level associations; edge thickness is proportional to Mantel r values, and edge color reflects statistical significance (dark purple: p < 0.001; medium purple: p < 0.01; light purple: p < 0.05; grey: p ≥ 0.05).

Similar results were observed when participants were stratified according to cumulative lifetime exposure. TEF expression was lower in the high-exposure group (p = 0.0374), whereas CLOCK showed a borderline reduction (p = 0.0674). No significant differences were detected for PER1 (p = 0.6847) or PER3 (p = 0.2384).

Stratification according to recent night-shift activity during the previous six months did not reveal significant differences for CLOCK (p = 0.3926), TEF (p = 0.3275), PER1 (p = 0.3731), or PER3 (p = 0.0991). Collectively, differences in gene expression were more evident across duration and cumulative exposure strata than according to recent exposure status.

To assess whether gene expression profiles could discriminate exposure categories, multivariable logistic regression models were constructed using duration of night-shift work, cumulative exposure, and recent exposure as outcomes (see **Supplementary Table 7**, and **Figure 3B**). The model based on duration of exposure showed good apparent discrimination (AUC = 0.744; 95% CI: 0.600–0.888; p = 0.0053) and significantly improved model fit compared with the intercept-only model (likelihood ratio p = 0.0457), with an overall classification accuracy of 68.9%.

Models based on cumulative exposure (AUC = 0.713; 95% CI: 0.561–0.864; p = 0.0158) and recent exposure (AUC = 0.709; 95% CI: 0.558–0.859; p = 0.0179) showed moderate discrimination, although neither significantly improved model fit relative to the null model (likelihood ratio p = 0.0879 and p = 0.2051, respectively). No individual gene was significantly associated with exposure category in these models.

Correlation analyses revealed differences in correlation structure across exposure strata (see **Supplementary Tables 8**-**S13**, and **Figure 3C**). In workers with shorter exposure duration, the strongest association was observed between PER1 and PER3 (ρ = 0.73, p < 0.001), whereas CLOCK showed weaker correlations with the other genes. In the long-exposure group, significant correlations remained detectable between PER1 and PER3 (ρ = 0.45, p = 0.026) and between TEF and CLOCK (ρ = 0.55, p = 0.004), while several additional pairwise correlations were attenuated.

Similar differences were observed across cumulative exposure strata. In the low-exposure group, the strongest association involved PER1 and PER3 (ρ = 0.69, p < 0.001), whereas in the high-exposure group stronger correlations were observed between CLOCK and TEF (ρ = 0.60, p = 0.001), TEF and PER1 (ρ = 0.41, p = 0.040), and PER1 and PER3 (ρ = 0.49, p = 0.011).

Analysis according to recent exposure status also identified distinct correlation patterns. In workers without recent night-shift activity, PER1 and PER3 remained strongly correlated (ρ = 0.53, p = 0.005), together with a moderate association between CLOCK and TEF (ρ = 0.50, p = 0.009). In workers reporting recent night-shift activity, the strongest correlation was observed between CLOCK and TEF (ρ = 0.70, p < 0.001). Mantel tests showed significant associations across all exposure strata (see **Supplementary Tables 8**-**S13**), supporting the presence of structured correlation patterns under each exposure condition.

### 3.5 YY1 expression differs according to night-shift exposure and improves the discriminatory performance of peripheral transcriptional signatures

Peripheral expression of the non-canonical circadian-associated transcriptional regulator YY1 was evaluated according to occupational schedule characteristics (**Figures 4A–F**). YY1 expression was significantly lower in NSW than in DSW (p = 0.0019) (**Figure 4A**). Within the NSW cohort, YY1 expression also differed according to exposure metrics (**Figure 4B**). Workers with long-term night-shift exposure showed lower YY1 expression than those with shorter exposure duration (p = 0.0333). Similarly, YY1 expression was lower in the high cumulative exposure group than in the low exposure group (p = 0.0315). No significant differences were observed according to recent night-shift activity.

**Figure 4:**
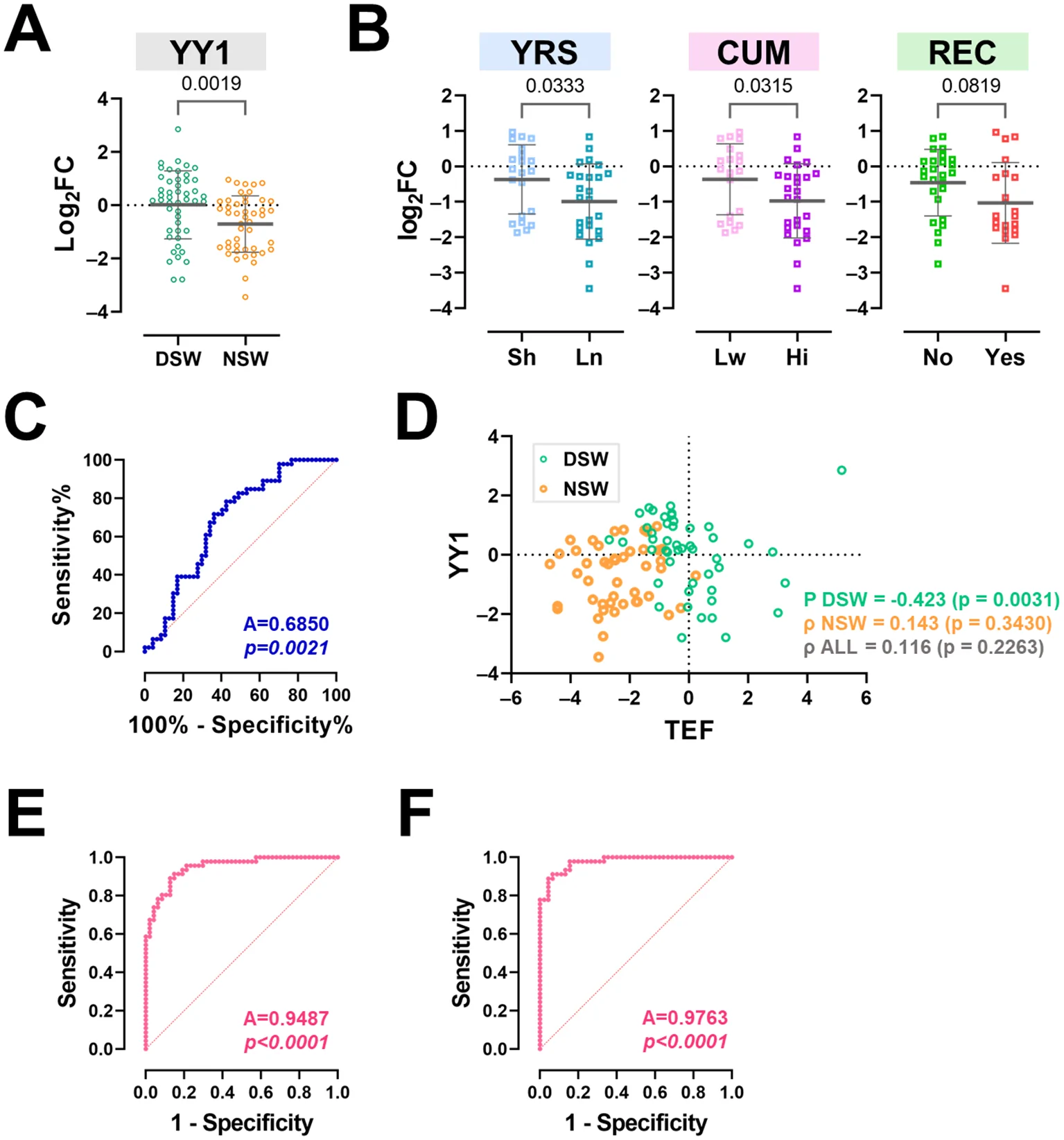
YY1 expression patterns, exposure stratification, and predictive performance in healthcare workers. **(A)** Dot plots showing RT-qPCR-derived log2 fold-change (Log2FC) expression of YY1 in day-shift workers (DSW; n = 47) and night-shift workers (NSW; n = 46). Each dot represents an individual participant. Mean ± SD are shown within each group, and exact p-values are reported for between-group comparisons. **(B)** Dot plots showing YY1 expression in NSW (n = 46) stratified according to occupational exposure characteristics. The left panel shows years of night-shift exposure (short: n = 21; long: n = 25), the middle panel shows cumulative exposure (low: n = 20; high: n = 26), and the right panel shows recent exposure status (no exposure: n = 26; exposure: n = 20). Each dot represents an individual participant. Mean ± SD are shown within each group, and exact p-values are reported for between-group comparisons. **(C)** Receiver operating characteristic (ROC) curve evaluating the ability of YY1 expression to discriminate between DSW and NSW. Area under the curve (AUC) values and corresponding p-values are reported. **(D)** Correlation analysis between YY1 and TEF expression levels. Each point represents an individual participant (DSW and NSW shown separately). Spearman correlation coefficients (ρ) and corresponding p-values are indicated within the panel. **(E)** ROC curve derived from a multivariable logistic regression model including YY1 and TEF expression levels. Area under the curve (AUC) values and corresponding p-values are reported. **(F)** ROC curve derived from a multivariable logistic regression model including CLOCK, TEF, PER1, PER3, and YY1 expression levels. Area under the curve (AUC) values and corresponding p-values are reported. Statistical significance was defined as p < 0.05.

Exploratory ROC analysis showed that YY1 discriminated between DSW and NSW with moderate accuracy (AUC = 0.6850; 95% CI: 0.5754–0.7946; p = 0.0021) (**Figure 4C**). To examine relationships between YY1 and core clock gene expression, correlation analyses were performed between YY1 and TEF (**Figure 4D**). In DSW, YY1 was inversely correlated with TEF (ρ = −0.423, p = 0.0031). No significant correlation was observed in NSW (ρ = 0.143, p = 0.3430) or in the overall cohort (ρ = 0.116, p = 0.2663).

Multivariable logistic regression models were then used to assess the contribution of YY1 to exposure classification (see **Supplementary Tables 14**, **S15**; and **Figures 4E, F**). In a reduced model including TEF and YY1, both variables were independently associated with shift-work status. TEF showed the strongest inverse association (OR = 0.1196; 95% CI: 0.03975–0.2602; p < 0.0001), while YY1 also remained significant (OR = 0.3971; 95% CI: 0.1947–0.7254; p = 0.0050). The model showed excellent apparent discrimination between DSW and NSW (AUC = 0.9487; 95% CI: 0.9084–0.9889; p < 0.0001) (**Figure 4E**).

When YY1 was added to the model including CLOCK, TEF, PER1, and PER3, apparent in-sample discrimination increased further (AUC = 0.9763; 95% CI: 0.9524–1.000; p < 0.0001) (**Figure 4F**). Within the multigene model, YY1 (OR = 0.1435; 95% CI: 0.03244–0.4023; p = 0.0017), TEF (OR = 0.0237; p = 0.0001), and CLOCK (OR = 11.66; p = 0.0095) were independently associated with group status, whereas PER1 and PER3 were not. Together, these analyses indicate that YY1 expression differs according to night-shift exposure and provides additional discriminatory information when combined with peripheral clock gene expression profiles.

### 3.6 Breast cancer status is not associated with additional alterations in peripheral clock genes expression among night-shift workers

To explore whether a history of breast cancer (BC) was associated with further differences in peripheral clock gene expression, transcript levels were compared among DSW, NSW without BC, and the subgroup of NSW with a previous diagnosis of BC (n = 5) (see **Supplementary Figure 1A**).

The differences previously observed between DSW and NSW were largely maintained in this analysis. CLOCK expression was lower in NSW than in DSW, although the comparison did not remain significant after adjustment for multiple testing (adjusted p = 0.1190). No significant differences were observed between BC and either DSW or NSW (all p > 0.18). TEF expression remained significantly lower in NSW than in DSW (adjusted p < 0.0001), whereas BC did not differ significantly from either group (DSW: p = 0.9300; NSW: p = 0.0985). Similarly, PER1 expression was reduced in NSW relative to DSW (p = 0.0012), while BC showed no significant differences compared with either reference group (all p > 0.78). PER3 expression did not differ across groups (all p > 0.94). Consistent results were observed for the composite CGES (see **Supplementary Figure 1B**). CGES values were lower in NSW than in DSW (adjusted p = 0.0001), whereas BC did not differ significantly from either DSW (p = 0.2372) or NSW (p > 0.9999).

## 4 Discussion

In this study, we investigated whether chronic night-shift work is associated with alterations in peripheral circadian transcriptional programs (TEF, CLOCK, PER1, PER3), and expression of the non-canonical transcriptional regulator YY1 in female healthcare workers. These alterations were more strongly associated with long-term and cumulative exposure than with recent shift activity, suggesting that peripheral transcriptional differences may be more closely related to longer-term occupational exposure than to recent night-shift involvement. Collectively, these findings support the presence of peripheral blood transcriptional patterns associated with occupational chronodisruption.

TEF showed the most pronounced differential expression, followed by PER1 and CLOCK. Previous studies have reported that night-shift work and light-at-night exposure can modulate peripheral clock gene expression, both in blood-derived cells and other tissues (21, 22, 24, 50–52). However, most human studies have focused on established core-clock genes. By contrast, PAR bZIP transcription factors such as TEF remained less explored in occupational exposures (53).

TEF participates in circadian transcriptional regulation and photic entrainment pathways (54, 55). Experimental evidence suggests that TEF contributes to light-responsive transcriptional regulation and D-box-mediated signaling, acting as an intermediary between environmental light stimuli and downstream circadian gene expression (56, 57). Although exploratory, our findings suggest that TEF may represent a particularly sensitive peripheral indicator of chronic circadian disruption. This is mechanistically plausible, as TEF operates downstream of the core clock and regulates circadian output programs involved in immune and metabolic pathways (58–61). Importantly, TEF may reflect circadian output failure rather than core oscillator disruption, consistent with its strong discriminatory performance observed in our ROC analyses (62).

PER3 showed a distinct pattern, with no significant differential expression between groups, yet contributing to multivariable and network-level analyses. This dissociation suggests a role beyond transcriptional amplitude regulation (63–65). Unlike PER1, which is more responsive to acute entrainment signals, PER3 has been linked to sleep homeostasis and inter-individual circadian vulnerability (66). Its behavior in PCA and clustering analyses suggests that PER3 may capture higher-order network organization rather than individual gene deregulation (67). Nevertheless, this interpretation remains exploratory.

Our data support the hypothesis that circadian disruption in occupational settings accumulates over time. The stronger association with cumulative and long-term exposure, compared to recent exposure, is consistent with persistent differences in peripheral blood transcriptional profiles associated with chronic night-shift work (68). In line with this hypothesis, experimental evidence supports progressive epigenetic and transcriptional changes under repeated circadian misalignment across peripheral tissues, including blood specimens (69–73). Importantly, although all samples were collected under standardized morning conditions, identical clock time does not necessarily correspond to the same biological circadian phase, particularly in night-shift workers. The absence of direct measures of internal circadian phase prevents discrimination between true transcriptional reprogramming and phase-related shifts in gene expression, both of which may contribute to the observed differences (74).

The multigene circadian signature showed excellent apparent in-sample discrimination between DSW and NSW, but lower performance in stratifying exposure intensity within NSW. This indicates that the signature primarily captures the presence of chronic shift-work exposure rather than fine-grained exposure heterogeneity. Residual variability likely reflects inter-individual factors such as chronotype, adaptation strategies, and genetic susceptibility, which might be not fully captured here (75–77).

Network analyses revealed that DSW exhibited a tightly coordinated transcriptional architecture, while NSW showed reduced gene–gene connectivity, particularly involving CLOCK-related interactions. These findings suggest that night-shift exposure may affect not only the expression of individual circadian genes but also their coordinated regulation (78, 79).

YY1, a non-canonical transcriptional regulator, was significantly downregulated in NSW and associated with both long-term and cumulative exposure. Given its role in chromatin remodeling and transcriptional integration, YY1 might reflect epigenetic adaptation to sustained circadian misalignment (33–35). Importantly, YY1 showed functional integration within the circadian network in DSW, where it correlated inversely with TEF. This relationship was lost in NSW, suggesting decoupling of transcriptional regulatory circuits under chronic circadian stress (36). This indicates that alterations associated with night-shift work could extend beyond canonical clock genes to involve multilayered transcriptional organization (3, 80, 81). Mechanistically, YY1 may act as a hub linking circadian regulation, chromatin remodeling, and inflammatory pathways, complementing TEF-driven output signaling (32, 82). Still, the very high discriminatory performance observed in the multigene models should be interpreted cautiously, as it may partly reflect the relative homogeneity of the study population and potential model overfitting inherent to exploratory analyses. External validation would be further required to determine the robustness, reproducibility, and generalizability of these findings.

The exploratory breast cancer subgroup analysis should be interpreted cautiously due to the very small sample size. Nevertheless, preservation of the night-shift-associated transcriptional pattern suggests relative stability of this circadian signature even in individuals with a prior cancer diagnosis, which contrasts with several transcriptomic studies reporting additional circadian gene alterations in night-shift workers with breast cancer (13). Discrepancies with previous computational findings, particularly regarding CLOCK regulation, may reflect context-dependent transcriptional responses across systemic versus tumor-associated environments (25, 83, 84).

Overall, our findings support a model in which peripheral circadian transcription is associated to chronic night-shift work exposure. TEF emerges as the most sensitive marker of chronic occupational circadian strain, while PER3 reflects network-level regulatory structure. YY1 adds an epigenetic and unestablished regulatory element.

From a precision medicine perspective, transcriptional profiles of peripheral clock genes could contribute to individualized approaches for the management of occupational circadian disruption (51). Multigene models may contribute to the characterization of circadian-related strain (38). However, clinical translation remains premature, as it is unclear whether these signatures predict disease risk or are reversible. At present, their utility is primarily mechanistic and exposure-stratification-oriented, providing a framework for identifying workers with distinct molecular responses to chronic night-shift exposure (85, 86). Further integration of molecular signatures with individual characteristics (including chronotype, sleep behavior, cumulative shift-work exposure, and other clinical risk factors), may ultimately facilitate chronobiology-guided occupational medicine, supporting targeted preventive interventions, optimization of work schedules, and evaluation of strategies aimed at mitigating long-term health consequences associated with chronic circadian misalignment.

Nevertheless, it remains unresolved whether the observed transcriptional remodeling reflects maladaptive disruption or partial compensatory adaptation aimed at preserving peripheral synchrony under chronic stress conditions (41, 43, 51). Further longitudinal and mechanistic studies are required to establish temporal relationships and causality, and to define the reversibility and clinical significance of the observed molecular alterations.

## 5 Strengths and limitations

This study has several strengths. It combines targeted analysis of selected circadian genes with multivariable and network-based approaches, allowing a multilevel characterization of peripheral transcriptional alterations associated with occupational night-shift work. The study population is relatively homogeneous, consisting of female healthcare workers with well-characterized occupational histories, which reduces heterogeneity related to sex and occupational background. Adjustment for multiple demographics, reproductive, lifestyle, and sleep-related covariates reduces, although does not eliminate, the risk of major confounding. Standardized blood collection procedures and fixed morning sampling times helped minimize pre-analytical and circadian phase–related variability. Finally, the inclusion of both canonical circadian genes and the non-canonical regulator YY1 provides a more comprehensive view of peripheral circadian transcriptional regulation under occupational circadian stress.

Several limitations should be acknowledged. The cross-sectional design precludes inference about temporality and causality. Residual confounding remains possible, particularly from unmeasured determinants of circadian regulation such as chronotype, sleep timing variability, nocturnal light exposure, psychological stress, and behavioral adaptation strategies. Peripheral blood gene expression provides an accessible but incomplete proxy of systemic circadian regulation and may not reflect tissue-specific circadian dynamics. Although sampling was standardized to morning hours, internal circadian phase was not assessed, and factors such as chronotype, sleep characteristics on the preceding night, and recovery following recent night-shift exposure were not directly measured. These factors may have contributed to inter-individual variability in gene expression independent of exposure status. The sample size limited statistical power for subgroup analyses, particularly in the exploratory breast cancer subgroup. In addition, the absence of direct physiological circadian markers, such as melatonin or cortisol rhythmic profiling, limits the ability to directly infer systemic circadian alignment. The ROC and multivariable classification analyses were exploratory and were performed and evaluated within the same cohort, without internal or external validation. Importantly, these analyses assessed discrimination of occupational exposure status and were not designed or powered to predict future disease or other clinical outcomes. Therefore, the observed discriminatory performance, particularly for the multigene models including YY1, may be optimistic and potentially influenced by overfitting. Independent validation in larger cohorts will be required to establish the robustness and generalizability of these findings. Finally, external validity is limited, as the findings may not generalize to male workers, other occupational groups, or shift systems with different temporal structures or rotation patterns.

## 6 Conclusions

Chronic night-shift work in female healthcare workers is associated with alterations in peripheral circadian gene expression, involving both canonical clock genes and non-canonical regulatory factors such as YY1. TEF and YY1 appear particularly sensitive to differences in night-shift exposure in this cohort, whereas PER3 may reflect inter-individual variability and network-level organization of circadian regulation. Overall, these findings support further investigation regarding peripheral circadian transcriptional profiles as candidate molecular signatures of chronic occupational night-shift exposure. Although their clinical and predictive relevance remains to be established in longitudinal and independently validated cohorts, integration of these molecular patterns with clinical, behavioral, and chronobiological characteristics, including chronotype and cumulative shift-work exposure, could support more tailored preventive strategies and improve the application of chronobiology-based approaches to occupational health.

## Data Availability

The raw data supporting the conclusions of this article will be made available by the authors, without undue reservation.
