# Supplementary material for: Night-shift work is associated with peripheral clock gene dysregulation and circadian transcriptional network remodeling in female healthcare workers
Source: Front Endocrinol (Lausanne). 2026 Sep 24;17:1934310. doi: 10.3389/fendo.2026.1934310 (PMC13645566; doi:10.3389/fendo.2026.1934310)
Supplement: Supplementary file 1 [file SupplementaryFile1.docx]

Supplementary Material

**Night-shift work is associated with peripheral clock gene dysregulation and circadian transcriptional network remodeling in female healthcare workers**

Silvia Vivarelli^1,†,*^, Luca Falzone^2,†^, Francesca Simona Fiorino^1^, Federica Giambò^3^, Massimo Libra^2^, Concettina Fenga^1^

^1^Department of Biomedical and Dental Sciences, Morphological and Functional Imaging, Section of Occupational Medicine, University of Messina, Messina, Italy

^2^Department of Biomedical and Biotechnological Sciences, University of Catania, Catania, Italy

^3^Vita-Salute San Raffaele University, Milano, Italy

^*^Correspondence: Silvia Vivarelli, [silvia.vivarelli@unime.it](mailto:silvia.vivarelli@unime.it)

^†^Silvia Vivarelli and Luca Falzone contributed equally to this work

# 1 Supplementary Figures and Tables

## 1.1 Supplementary Tables

**Supplementary Table S1. Anthropometric and demographic characteristics of the study population**

| Variable | Total (n = 93) Mean (SD) | DSW (n = 47) Mean (SD) | NSW (n = 46) Mean (SD) | p-value |
| --- | --- | --- | --- | --- |
| Age (years) | 51.32 (8.12) | 51.64 (8.72) | 51.00 (7.55) | 0.707 |
| BMI (kg/m²) | 24.35 (4.90) | 24.26 (5.34) | 24.44 (4.47) | 0.857 |

Abbreviations: DSW, day-shift workers; NSW, night-shift workers; SD, standard deviation; BMI, body mass index. Statistical significance was set at p < 0.05.

**Supplementary Table S2. Occupational exposure characteristics among night-shift workers**

| Exposure domain | Category | N (%) |
| --- | --- | --- |
| Years of exposure | 1–12 years (short) | 21 (46) |
|  | 13–35 years (long) | 25 (54) |
| Cumulative night shifts | 96–999 (low) | 20 (43) |
|  | 1000–3018 (high) | 26 (57) |
| Recent night-shift activity (≥1 night shift in previous 6 months) (*) | No | 26 (57) |
|  | Yes | 20 (43) |

(*) Recent night-shift activity was defined as having performed at least one night shift during the six months preceding study enrollment. Among participants with recent activity (n = 20), the number of night shifts ranged from 3 to 8, with a mean of 5.4 ± 1.1 shifts.

**Supplementary Table S3. Lifestyle, reproductive, and sleep characteristics of the study population**

| Variable | Category | Total (n = 93) n (%) | DSW (n = 47) n (%) | NSW (n = 46) n (%) | p-value |
| --- | --- | --- | --- | --- | --- |
| Pregnancies | No | 26 (28) | 15 (32) | 11 (24) | 0.390 |
|  | Yes | 67 (72) | 32 (68) | 35 (76) |  |
| Menopause | No | 51 (55) | 27 (57) | 24 (52) | 0.609 |
|  | Yes | 42 (45) | 20 (43) | 22 (48) |  |
| Smoking | No | 51 (55) | 30 (64) | 21 (46) | 0.201 |
|  | Past | 16 (17) | 7 (15) | 9 (20) |  |
|  | Current | 26 (28) | 10 (21) | 16 (35) |  |
| Alcohol consumption | No | 32 (34) | 21 (45) | 11 (24) | **0.035** |
|  | Yes | 61 (66) | 26 (55) | 35 (76) |  |
| Exercise habits | No | 57 (61) | 32 (68) | 25 (54) | 0.174 |
|  | Yes | 36 (39) | 15 (32) | 21 (46) |  |
| Sleep satisfaction | Very good | 9 (10) | 8 (17) | 1 (2) | **0.004** |
|  | Good | 40 (43) | 20 (43) | 20 (43) |  |
|  | Bad | 36 (39) | 19 (40) | 17 (37) |  |
|  | Very bad | 8 (9) | 0 (0) | 8 (17) |  |
| Sleep duration (hours) | Mean (SD) | 6.01 (1.22) | 6.26 (1.31) | 5.76 (1.08) | 0.051 |

Abbreviations: DSW, day-shift workers; NSW, night-shift workers; SD, standard deviation. Statistical significance was set at p < 0.05; statistically significant p-values are reported in bold.

**Supplementary Table S4. Spearman correlation matrix and Mantel test results for clock genes, CGES, occupational, lifestyle, and clinical variables in healthcare workers**

| Variable | CLOCK | TEF | PER1 | PER3 | CGES | SLP_HRS | SLP_DIS | SMK | ALC | EXE | AGE | BMI | MEN | PAR | *r (Mantel)* | *p-value (Mantel)* |
| --- | --- | --- | --- | --- | --- | --- | --- | --- | --- | --- | --- | --- | --- | --- | --- | --- |
| CLOCK | 1 | **0.462 (4.6e-06)** | **0.481 (1.6e-06)** | 0.125 (0.239) | **0.667 (<1e-12)** | 0.087 (0.414) | -0.119 (0.263) | 0.118 (0.267) | 0.042 (0.696) | -0.057 (0.595) | -0.073 (0.493) | 0.173 (0.103) | -0.097 (0.365) | **-0.208 (0.050)** | *0.069* | *0.095* |
| TEF | **0.462 (4.6e-06)** | 1 | **0.642 (4.2e-12)** | **0.286 (0.006)** | **0.796 (<1e-20)** | **0.272 (0.008)** | **-0.330 (0.001)** | 0.071 (0.501) | -0.169 (0.106) | -0.066 (0.531) | 0.018 (0.865) | 0.098 (0.352) | -0.087 (0.407) | -0.048 (0.650) | *0.025* | *0.302* |
| PER1 | **0.481 (1.6e-06)** | **0.642 (4.2e-12)** | 1 | **0.508 (2.1e-07)** | **0.858 (<1e-26)** | 0.166 (0.112) | -0.147 (0.159) | **0.236 (0.023)** | -0.079 (0.450) | 0.116 (0.268) | 0.056 (0.595) | **0.230 (0.027)** | 0.035 (0.736) | -0.042 (0.687) | *0.093* | *0.058* |
| PER3 | 0.125 (0.239) | **0.286 (0.006)** | **0.508 (2.1e-07)** | 1 | **0.664 (<1e-12)** | 0.138 (0.188) | **-0.221 (0.033)** | 0.172 (0.100) | -0.096 (0.359) | 0.179 (0.086) | 0.010 (0.925) | 0.056 (0.595) | 0.013 (0.902) | -0.132 (0.209) | ***0.113*** | ***0.009*** |
| CGES | **0.667 (<1e-12)** | **0.796 (<1e-20)** | **0.858 (<1e-26)** | **0.664 (<1e-12)** | 1 | 0.204 (0.054) | **-0.259 (0.014)** | 0.176 (0.098) | -0.086 (0.419) | 0.084 (0.432) | -0.033 (0.755) | 0.168 (0.113) | -0.099 (0.352) | -0.165 (0.119) | ***0.109*** | ***0.033*** |
| SLP_HRS | 0.087 (0.414) | **0.272 (0.008)** | 0.166 (0.112) | 0.138 (0.188) | 0.204 (0.054) | 1 | **-0.617 (<1e-10)** | 0.011 (0.918) | 0.025 (0.814) | 0.042 (0.692) | **-0.289 (0.005)** | -0.083 (0.428) | **-0.300 (0.003)** | -0.012 (0.906) | ***0.082*** | ***0.034*** |
| SLP_DIS | -0.119 (0.263) | **-0.330 (0.001)** | -0.147 (0.159) | **-0.221 (0.033)** | **-0.259 (0.014)** | **-0.617 (<1e-10)** | 1 | -0.172 (0.100) | 0.097 (0.356) | 0.003 (0.976) | 0.043 (0.685) | -0.055 (0.603) | 0.131 (0.211) | -0.159 (0.129) | *-0.021* | *0.694* |
| SMK | 0.118 (0.267) | 0.071 (0.501) | **0.236 (0.023)** | 0.172 (0.100) | 0.176 (0.098) | 0.011 (0.918) | -0.172 (0.100) | 1 | -0.026 (0.803) | -0.109 (0.299) | 0.050 (0.636) | 0.020 (0.850) | 0.047 (0.655) | 0.178 (0.088) | *0.025* | *0.206* |
| ALC | 0.042 (0.696) | -0.169 (0.106) | -0.079 (0.450) | -0.096 (0.359) | -0.086 (0.419) | 0.025 (0.814) | 0.097 (0.356) | -0.026 (0.803) | 1 | 0.018 (0.864) | 0.041 (0.700) | 0.028 (0.787) | -0.116 (0.269) | -0.098 (0.351) | *-0.011* | *0.608* |
| EXE | -0.057 (0.595) | -0.066 (0.531) | 0.116 (0.268) | 0.179 (0.086) | 0.084 (0.432) | 0.042 (0.692) | 0.003 (0.976) | -0.109 (0.299) | 0.018 (0.864) | 1 | -0.025 (0.814) | -0.116 (0.270) | **-0.233 (0.024)** | -0.114 (0.278) | *-0.031* | *0.948* |
| AGE | -0.073 (0.493) | 0.018 (0.865) | 0.056 (0.595) | 0.010 (0.925) | -0.033 (0.755) | **-0.289 (0.005)** | 0.043 (0.685) | 0.050 (0.636) | 0.041 (0.700) | -0.025 (0.814) | 1 | **0.246 (0.018)** | **0.783 (<1e-19)** | **0.225 (0.030)** | ***0.900*** | ***0.001*** |
| BMI | 0.173 (0.103) | 0.098 (0.352) | **0.230 (0.027)** | 0.056 (0.595) | 0.168 (0.113) | -0.083 (0.428) | -0.055 (0.603) | 0.020 (0.850) | 0.028 (0.787) | -0.116 (0.270) | **0.246 (0.018)** | 1 | 0.183 (0.079) | 0.072 (0.490) | ***0.421*** | ***0.001*** |
| MEN | -0.097 (0.365) | -0.087 (0.407) | 0.035 (0.736) | 0.013 (0.902) | -0.099 (0.352) | **-0.300 (0.003)** | 0.131 (0.211) | 0.047 (0.655) | -0.116 (0.269) | **-0.233 (0.024)** | **0.783 (<1e-20)** | 0.183 (0.079) | 1 | **0.294 (0.004)** | ***0.476*** | ***0.001*** |
| PAR | **-0.208 (0.050)** | -0.048 (0.650) | -0.042 (0.687) | -0.132 (0.209) | -0.165 (0.119) | -0.012 (0.906) | -0.159 (0.129) | 0.178 (0.088) | -0.098 (0.351) | -0.114 (0.278) | **0.225 (0.030)** | 0.072 (0.490) | **0.294 (0.004)** | 1 | *0.051* | ***0.017*** |

Values represent Spearman correlation coefficients (ρ) with corresponding p-values in parentheses. The last two columns report Mantel test results assessing global network-level associations for each variable. Abbreviations: CGES, Clock Gene Expression Score; SLP_HRS, sleep duration; SLP_DIS, sleep dissatisfaction; SMK, smoking; ALC, alcohol consumption; EXE, physical exercise; AGE, age; BMI, body mass index; MEN, menopausal status; PAR, parity; Mantel r, Mantel correlation coefficient. Statistical significance was set at p < 0.05; statistically significant p-values are reported in bold.

**Supplementary Table S5. Spearman correlation matrix and Mantel test results for clock genes expression in day-shift workers**

| Variable | CLOCK | TEF | PER1 | PER3 | CGES | *r (Mantel)* | *p-value (Mantel)* |
| --- | --- | --- | --- | --- | --- | --- | --- |
| CLOCK | 1 | **0.333 (0.025)** | **0.532 (1.7e-04)** | 0.141 (0.357) | **0.600 (1.3e-05)** | ***0.721*** | ***0.001*** |
| TEF | **0.333 (0.025)** | 1 | **0.717 (1.4e-08)** | **0.542 (8.3e-05)** | **0.800 (4.3e-11)** | ***0.833*** | ***0.001*** |
| PER1 | **0.532 (1.7e-04)** | **0.717 (1.4e-08)** | 1 | **0.482 (6.1e-04)** | **0.887 (5.5e-16)** | ***0.851*** | ***0.001*** |
| PER3 | 0.141 (0.357) | **0.542 (8.3e-05)** | **0.482 (6.1e-04)** | 1 | **0.769 (6.7e-10)** | ***0.638*** | ***0.001*** |
| CGES | **0.600 (1.3e-05)** | **0.800 (4.3e-11)** | **0.887 (5.5e-16)** | **0.769 (6.7e-10)** | 1 | ***0.867*** | ***0.001*** |

Values represent Spearman correlation coefficients (ρ) with corresponding p-values in parentheses. The last two columns report Mantel test results assessing global network-level associations for each variable. Abbreviations: CGES, Clock Gene Expression Score. Statistical significance was set at p < 0.05; statistically significant p-values are reported in bold.

**Supplementary Table S6. Spearman correlation matrix and Mantel test results for clock genes expression in night-shift workers**

| Variable | CLOCK | TEF | PER1 | PER3 | CGES | *r (Mantel)* | *p-value (Mantel)* |
| --- | --- | --- | --- | --- | --- | --- | --- |
| CLOCK | 1 | **0.559 (6.7e-05)** | **0.322 (0.031)** | 0.070 (0.648) | **0.654 (1.1e-06)** | ***0.547*** | ***0.001*** |
| TEF | **0.559 (6.7e-05)** | 1 | **0.410 (0.0047)** | 0.154 (0.308) | **0.712 (4.2e-08)** | ***0.664*** | ***0.001*** |
| PER1 | **0.322 (0.031)** | **0.410 (0.0047)** | 1 | **0.586 (1.9e-05)** | **0.763 (1.1e-09)** | ***0.564*** | ***0.001*** |
| PER3 | 0.070 (0.648) | 0.154 (0.308) | **0.586 (1.9e-05)** | 1 | **0.633 (3.1e-06)** | ***0.555*** | ***0.001*** |
| CGES | **0.654 (1.1e-06)** | **0.712 (4.2e-08)** | **0.763 (1.1e-09)** | **0.633 (3.1e-06)** | 1 | ***0.799*** | ***0.001*** |

Values represent Spearman correlation coefficients (ρ) with corresponding p-values in parentheses. The last two columns report Mantel test results assessing global network-level associations for each variable. Abbreviations: CGES, Clock Gene Expression Score. Statistical significance was set at p < 0.05; statistically significant p-values are reported in bold.

**Supplementary Table S7. Multivariable logistic regression models and ROC analysis for clock genes expression signatures predicting occupational exposure in night-shift workers**

| Outcome | Variable | β (SE) | OR (95% CI) | p-value | AUC (95% CI) | AUC p-value | Model p-value |
| --- | --- | --- | --- | --- | --- | --- | --- |
| YEARS | CLOCK | -0.502 (0.5237) | 0.61 (0.20–1.62) | 0.338 | **0.744 (0.600–0.888)** | **0.005** | **0.046** |
|  | TEF | -0.479 (0.3717) | 0.62 (0.28–1.25) | 0.198 |  |  |  |
|  | PER1 | -0.452 (0.5691) | 0.64 (0.19–1.97) | 0.427 |  |  |  |
|  | PER3 | 0.692 (0.4439) | 2.00 (0.87–5.21) | 0.119 |  |  |  |
| CUM | CLOCK | -0.304 (0.5122) | 0.74 (0.25–1.95) | 0.552 | **0.713 (0.561–0.864)** | **0.016** | 0.088 |
|  | TEF | -0.467 (0.3667) | 0.63 (0.29–1.26) | 0.203 |  |  |  |
|  | PER1 | -0.406 (0.5519) | 0.67 (0.21–2.00) | 0.462 |  |  |  |
|  | PER3 | 0.775 (0.4415) | 2.17 (0.96–5.68) | 0.079 |  |  |  |
| REC | CLOCK | -0.843 (0.5186) | 0.43 (0.14–1.12) | 0.104 | **0.709 (0.558–0.859)** | **0.018** | 0.205 |
|  | TEF | 0.471 (0.3549) | 1.60 (0.81–3.34) | 0.184 |  |  |  |
|  | PER1 | 0.172 (0.5503) | 1.19 (0.38–3.58) | 0.754 |  |  |  |
|  | PER3 | 0.400 (0.3985) | 1.49 (0.71–3.54) | 0.316 |  |  |  |

Values represent multivariable logistic regression coefficients (β) with standard errors (SE) in parentheses and odds ratios (OR) with 95% confidence intervals (CI). Statistically significant p-values (p < 0.05) are shown in bold. Model performance is reported using receiver operating characteristic (ROC) analysis. Area under the curve (AUC) values with 95% CI are shown. AUC p-values indicate discrimination significance. Model p-values correspond to overall model significance. Abbreviations: YEARS, years of exposure; CUM, cumulative exposure; REC, recent exposure.

**Supplementary Table S8. Spearman correlation matrix and Mantel test results for clock genes expression in night-shift workers stratified by years of exposure (short group)**

| Variable | CLOCK | TEF | PER1 | PER3 | *r (Mantel)* | *p-value (Mantel)* |
| --- | --- | --- | --- | --- | --- | --- |
| CLOCK | 1 | **0.456 (0.043)** | 0.362 (0.116) | 0.236 (0.316) | ***0.424*** | ***0.002*** |
| TEF | **0.456 (0.043)** | 1 | **0.435 (0.049)** | 0.225 (0.328) | ***0.384*** | ***0.002*** |
| PER1 | 0.362 (0.116) | **0.435 (0.049)** | 1 | **0.725 (2.0e-04)** | ***0.690*** | ***0.001*** |
| PER3 | 0.236 (0.316) | 0.225 (0.328) | **0.725 (2.0e-04)** | 1 | ***0.692*** | ***0.001*** |

Values represent Spearman correlation coefficients (ρ) with corresponding p-values in parentheses. Statistically significant correlations (p < 0.05) are shown in bold. The last two columns report Mantel test results assessing global network-level associations for each gene. Mantel p-values < 0.05 were considered statistically significant.

**Supplementary Table S9. Spearman correlation matrix and Mantel test results for clock genes expression in night-shift workers stratified by years of exposure (long group)**

| Variable | CLOCK | TEF | PER1 | PER3 | *r (Mantel)* | *p-value (Mantel)* |
| --- | --- | --- | --- | --- | --- | --- |
| CLOCK | 1 | **0.552 (0.0042)** | 0.377 (0.063) | 0.026 (0.901) | ***0.527*** | ***0.001*** |
| TEF | **0.552 (0.0042)** | 1 | 0.343 (0.093) | 0.209 (0.315) | ***0.761*** | ***0.001*** |
| PER1 | 0.377 (0.063) | 0.343 (0.093) | 1 | **0.445 (0.026)** | ***0.558*** | ***0.001*** |
| PER3 | 0.026 (0.901) | 0.209 (0.315) | **0.445 (0.026)** | 1 | ***0.546*** | ***0.001*** |

Values represent Spearman correlation coefficients (ρ) with corresponding p-values in parentheses. Statistically significant correlations (p < 0.05) are shown in bold. The last two columns report Mantel test results assessing global network-level associations for each gene. Mantel p-values < 0.05 were considered statistically significant.

**Supplementary Table S10. Spearman correlation matrix and Mantel test results for clock genes expression in night-shift workers stratified by cumulative exposure (low group)**

| Variable | CLOCK | TEF | PER1 | PER3 | *r (Mantel)* | *p-value (Mantel)* |
| --- | --- | --- | --- | --- | --- | --- |
| CLOCK | 1 | 0.398 (0.091) | 0.307 (0.201) | 0.126 (0.606) | ***0.386*** | ***0.001*** |
| TEF | 0.398 (0.091) | 1 | 0.385 (0.094) | 0.114 (0.631) | ***0.359*** | ***0.004*** |
| PER1 | 0.307 (0.201) | 0.385 (0.094) | 1 | **0.690 (7.6e-04)** | ***0.699*** | ***0.001*** |
| PER3 | 0.126 (0.606) | 0.114 (0.631) | **0.690 (7.6e-04)** | 1 | ***0.683*** | ***0.001*** |

Values represent Spearman correlation coefficients (ρ) with corresponding p-values in parentheses. Statistically significant correlations (p < 0.05) are shown in bold. The last two columns report Mantel test results assessing global network-level associations for each gene. Mantel p-values < 0.05 were considered statistically significant.

**Supplementary Table S11. Spearman correlation matrix and Mantel test results for clock genes expression in night-shift workers stratified by cumulative exposure (high group)**

| Variable | CLOCK | TEF | PER1 | PER3 | *r (Mantel)* | *p-value (Mantel)* |
| --- | --- | --- | --- | --- | --- | --- |
| CLOCK | 1 | **0.595 (0.0014)** | **0.444 (0.023)** | 0.105 (0.610) | ***0.571*** | ***0.001*** |
| TEF | **0.595 (0.0014)** | 1 | **0.405 (0.040)** | 0.270 (0.181) | ***0.768*** | ***0.001*** |
| PER1 | **0.444 (0.023)** | **0.405 (0.040)** | 1 | **0.490 (0.011)** | ***0.589*** | ***0.001*** |
| PER3 | 0.105 (0.610) | 0.270 (0.181) | **0.490 (0.011)** | 1 | ***0.541*** | ***0.001*** |

Values represent Spearman correlation coefficients (ρ) with corresponding p-values in parentheses. Statistically significant correlations (p < 0.05) are shown in bold. The last two columns report Mantel test results assessing global network-level associations for each gene. Mantel p-values < 0.05 were considered statistically significant.

**Supplementary Table S12. Spearman correlation matrix and Mantel test results for clock genes expression in night-shift workers stratified by recent exposure (no exposure group)**

| Variable | CLOCK | TEF | PER1 | PER3 | *r (Mantel)* | *p-value (Mantel)* |
| --- | --- | --- | --- | --- | --- | --- |
| CLOCK | 1 | **0.501 (0.009)** | 0.374 (0.060) | 0.198 (0.332) | ***0.571*** | ***0.001*** |
| TEF | **0.501 (0.009)** | 1 | 0.344 (0.086) | 0.129 (0.530) | ***0.724*** | ***0.001*** |
| PER1 | 0.374 (0.060) | 0.344 (0.086) | 1 | **0.531 (0.005)** | ***0.621*** | ***0.001*** |
| PER3 | 0.198 (0.332) | 0.129 (0.530) | **0.531 (0.005)** | 1 | ***0.707*** | ***0.001*** |

Values represent Spearman correlation coefficients (ρ) with corresponding p-values in parentheses. Statistically significant correlations (p < 0.05) are shown in bold. The last two columns report Mantel test results assessing global network-level associations for each gene. Mantel p-values < 0.05 were considered statistically significant.

**Supplementary Table S13. Spearman correlation matrix and Mantel test results for clock genes expression in night-shift workers stratified by recent exposure (exposed group)**

| Variable | CLOCK | TEF | PER1 | PER3 | *r (Mantel)* | *p-value (Mantel)* |
| --- | --- | --- | --- | --- | --- | --- |
| CLOCK | 1 | **0.702 (8.1e-04)** | 0.295 (0.221) | -0.021 (0.932) | ***0.516*** | ***0.001*** |
| TEF | **0.702 (8.1e-04)** | 1 | 0.338 (0.145) | -0.008 (0.975) | ***0.693*** | ***0.001*** |
| PER1 | 0.295 (0.221) | 0.338 (0.145) | 1 | **0.642 (0.0023)** | ***0.433*** | ***0.001*** |
| PER3 | -0.021 (0.932) | -0.008 (0.975) | **0.642 (0.0023)** | 1 | ***0.287*** | ***0.015*** |

Values represent Spearman correlation coefficients (ρ) with corresponding p-values in parentheses. Statistically significant correlations (p < 0.05) are shown in bold. The last two columns report Mantel test results assessing global network-level associations for each gene. Mantel p-values < 0.05 were considered statistically significant.

**Supplementary Table S14. Multiple logistic regression analysis of exposure status prediction based on TEF and YY1 expression**

| Variable | β (SE) | OR (95% CI) | p-value | AUC (95% CI) | AUC p-value | Model p-value | Interpretation |
| --- | --- | --- | --- | --- | --- | --- | --- |
| TEF | -2.124 (0.46930) | 0.1196 (0.03975–0.2602) | **<0.0001** | 0.9487 (0.9084–0.9889) | **<0.0001** | **<0.0001** | Inverse association  (DSW-predictive) |
| YY1 | -0.9235 (0.32930) | 0.3971 (0.1947–0.7254) | **0.0050** | — | — | — | Moderate inverse association  (DSW-predictive) |

β coefficients are shown with standard errors (SE). Odds ratios (OR) are presented with 95% confidence intervals (CI). Statistically significant associations (p < 0.05) are highlighted in bold.

**Supplementary Table S15. Multiple logistic regression analysis of exposure status prediction based on all clock genes and YY1 expression**

| Variable | β (SE) | OR (95% CI) | p-value | AUC (95% CI) | AUC p-value | Model p-value | Interpretation |
| --- | --- | --- | --- | --- | --- | --- | --- |
| CLOCK | 2.456 (0.94720) | 11.66 (2.336–106.7) | **0.0095** | 0.9763 (0.9524–1.000) | **<0.0001** | **<0.0001** | Strong positive association  (NSW-predictive) |
| TEF | -3.742 (0.97860) | 0.0237 (0.002204–0.1115) | **0.0001** | — | — | — | Strong inverse association  (DSW-predictive) |
| PER1 | -0.8319 (0.99820) | 0.4352 (0.05358–2.915) | 0.4046 | — | — | — | Not significant |
| PER3 | 1.104 (0.57080) | 3.015 (1.145–11.27) | 0.0532 | — | — | — | Borderline |
| YY1 | -1.942 (0.61900) | 0.1435 (0.03244–0.4023) | **0.0017** | — | — | — | Inverse association  (DSW-predictive) |

β coefficients are shown with standard errors (SE). Odds ratios (OR) are presented with 95% confidence intervals (CI). Statistically significant associations (p < 0.05) are highlighted in bold.

# 2 Supplementary Figures

**Supplementary Figure S1.** **Clock genes expression profiles in healthcare workers with and without breast cancer. (A)** Dot plots showing RT-qPCR-derived log2 fold-change (Log2FC) expression of core clock genes (CLOCK, TEF, PER1, and PER3) in day-shift workers (DSW; n = 47), night-shift workers (NSW; n = 46), and night-shift workers with a history of breast cancer (BC; n = 5). Each dot represents an individual participant. Mean ± SD are shown within each group, and exact p-values are reported for pairwise comparisons. **(B)** Dot plots showing the Clock Gene Expression Score (CGES; arbitrary units) in DSW, NSW, and BC groups. Mean ± SD and exact p-values are displayed as in panel (A). Statistical significance was defined as p < 0.05.
